# Inter-Layer Coupling Induced Valence Band Edge Shift in Mono- to Few-Layer MoS_2_

**DOI:** 10.1038/srep40559

**Published:** 2017-01-13

**Authors:** Daniel J. Trainer, Aleksei V. Putilov, Cinzia Di Giorgio, Timo Saari, Baokai Wang, Mattheus Wolak, Ravini U. Chandrasena, Christopher Lane, Tay-Rong Chang, Horng-Tay Jeng, Hsin Lin, Florian Kronast, Alexander X. Gray, Xiaoxing X. Xi, Jouko Nieminen, Arun Bansil, Maria Iavarone

**Affiliations:** 1 Physics Department, Temple University, Philadelphia PA 19122, USA; 2 Department of Physics, Tampere University of Technology, Tampere, Finland; 3 Physics Department, Northeastern University, Boston MA 02115, USA; 4 Department of Physics, National Tsing Hua University, Hsinchu 30013, Taiwan; 5 Institute of Physics, Academia Sinica, Taipei 11529, Taiwan; 6 Centre for Advanced 2D Materials and Graphene Research Centre, National University of Singapore, 117546 Singapore; 7 Department of Physics, National University of Singapore, 117546 Singapore; 8 Helmholtz-Zentrum Berlin für Materialien und Energie, Albert-Einstein Straße 15, 12489 Berlin, Germany

## Abstract

Recent progress in the synthesis of monolayer MoS_2_, a two-dimensional direct band-gap semiconductor, is paving new pathways toward atomically thin electronics. Despite the large amount of literature, fundamental gaps remain in understanding electronic properties at the nanoscale. Here, we report a study of highly crystalline islands of MoS_2_ grown via a refined chemical vapor deposition synthesis technique. Using high resolution scanning tunneling microscopy and spectroscopy (STM/STS), photoemission electron microscopy/spectroscopy (PEEM) and μ-ARPES we investigate the electronic properties of MoS_2_ as a function of the number of layers at the nanoscale and show in-depth how the band gap is affected by a shift of the valence band edge as a function of the layer number. Green’s function based electronic structure calculations were carried out in order to shed light on the mechanism underlying the observed bandgap reduction with increasing thickness, and the role of the interfacial Sulphur atoms is clarified. Our study, which gives new insight into the variation of electronic properties of MoS_2_ films with thickness bears directly on junction properties of MoS_2_, and thus impacts electronics application of MoS_2_.

Two-dimensional (2D) materials have recently attracted attention for their potential applications in electronics and optoelectronics devices. Although graphene is the workhorse 2D material^1^ for the richness of physics that it displays^2^,^3^ and its high carrier mobility^4^, the lack of a bandgap limits its application in the semiconducting industry. 2D transition metal dichalcogenides (TMDs), with the general formula MX_2_ (where M = Mo, W; X = Se, S, Te) are layered materials typically composed of planar sheets with strong in-plane bonds and with layers weakly bound by van der Waals interactions, facilitating the isolation of single or few layers similar to graphene. In the bulk form the physical properties of TMDs are diverse ranging from insulators such as HfS_2_, to semiconductors such as MoS_2_ and WS_2_, to semimetals such as WTe_2_ and TiSe_2_, and to true metals such as NbSe_2_ and VSe_2_. Their phase diagrams under temperature, doping and pressure are very rich, and display instabilities like charge density waves with commensurate, incommensurate, short-range correlations and chiral order^5^,^6^,^7^,^8^, superconductivity^9^, excitonic condensation^10^ and Mott-insulator transitions^11^. More remarkably, their properties at the level of a monolayer or a few atomic layers can be strongly modified. In the case of MoS_2_, there is a transition from an indirect band gap in the bulk to a direct band gap at monolayer^12^ that can be tuned by functionalization or purposeful tweaking, opening up the possibility of flexible electronics applications^13^,^14^ and field effect transistors^15^,^16^. Unlike graphene, monolayer MoS_2_ lacks spatial inversion symmetry and has strong spin-orbit coupling originating from the *d*-orbitals of the heavy transition metal atom. This coupled with a large direct band gap induces spin-split valence bands around the K-point^17^, making MoS_2_ a promising material for spin/valley electronics.

Among the most interesting properties of 2D-TMDs is the tunability of the electronic properties as a function of layer thickness^18^,^19^, stress^20^,^21^, defects such as vacancies, and doping and intercalation^12^,^22^,^23^,^24^. Several studies have been performed on atomically thin TMDs to characterize their electronic and structural properties. A majority of these studies include photoluminescence experiments to determine optical bandgaps^12^,^20^,^21^,^25^ and high-resolution transmission microscopy to determine the crystal structure^26^,^27^,^28^. Other advanced surface science techniques such as angle-resolved photoemission spectroscopy (ARPES)^18^ and STM/STS^29^,^30^,^31^ have been used to probe the electronic properties of 2D TMDs. However, due to the stringent requirements of high-quality samples with large single-crystalline domains, high uniformity, clean, atomically flat and conductive substrates, there is paucity of high-quality STM/STS data on intrinsic electronic properties of MX_2_ layers at the atomic scale.

In this work we report a systematic study of the evolution of electronic properties of ultrathin MoS_2_ films as a function of layer number. We employ a comprehensive approach, which combines STM/STS, PEEM and μ-ARPES measurements. In this way we address nanoscale properties of MoS_2_ and adduce fundamental information relevant for applications. The MoS_2_ samples were directly grown on a graphite substrate by the chemical vapour deposition (CVD) method^32^ to avoid contamination introduced by chemical transfer. The layer-dependent tunneling and PEEM spectra are modeled using Green’s function techniques within a tight-binding (TB) framework utilizing density functional theory (DFT) to unfold the mechanism responsible for producing reduction in the bandgap with film thickness.

## Results

### Structural and scanning tunneling microscopy/spectroscopy characterization

STM/STS allows us to measure the intrinsic one-electron quasiparticle gap and correlate it with the local environment at the atomic scale. The vulnerability of atomically thin layers of 2D materials to environmental disturbances has prompted an ongoing search for substrates that can support the material without perturbing its electronic structure. Graphite substrates are found to be by far the least invasive, making it possible to access the intrinsic low-energy spectrum of 2D materials via STM/STS^33^,^34^.

**Figure 1:**
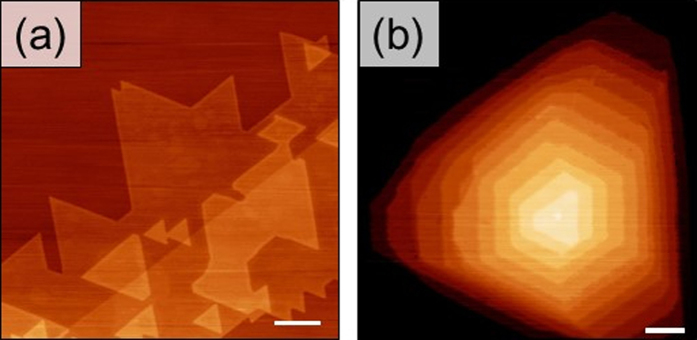
AFM MoS_2_ film’s morphology. Large-scale topography of stacked monolayer MoS_2_ films of varying morphologies on HOPG substrate. (**a**) and (**b**) show AFM images of stacked MoS_2_ triangular and hexagonal structures, respectively. Scale bars represent 200 nm.

Few layer MoS_2_ films were prepared using the ambient pressure chemical vapor deposition (APCVD) technique on highly oriented pyrolytic graphite (HOPG) substrates with solid MoO_3_ and S precursors^32^. This technique yields highly crystalline, stacked single-layer MoS_2_ domains as revealed by atomic force microscopy (AFM) (Fig. 1). The AFM images show typical film morphologies that vary between triangular and hexagonal structures as a consequence of the crystal structure of MoS_2_. In literature, it has been suggested that the triangular morphologies form as a result of the off-stoichiometric, local growth conditions that might cause the edges terminated by Mo (S), for example, to grow faster than those terminated by S (Mo)^35^. In this scenario, when Mo/S ratio corresponds to the stoichiometry of MoS_2_, the termination stability and the probability for the formation of two types of terminations would be similar, and this would result in similar growth rates that lead to hexagonal domains. The parallel edges of stacked MoS_2_ structures, in the samples studied, indicate that the vertical layers tend to grow with little lattice rotation between them.

**Figure 2:**
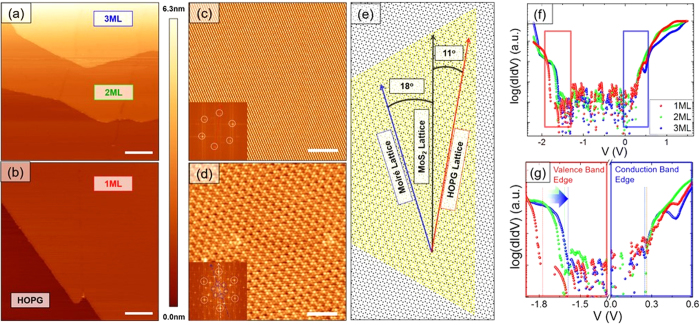
STM/STS characterization of MoS2 films. (**a**) and (**b**) STM topography showing one monolayer, two monolayer and three monolayer thick terraces of MoS_2_ (V = +1.75 V, I = 10 pA) where the underlying graphite can be seen in the bottom part of (**b**) (V = +3.0 V, I = 250 pA). The scale bar represents 50 nm. (**c**) and (**d**) show atomic resolution topographies of the HOPG substrate (V = +1.0 V, I = 200 pA) and the MoS_2_ monolayer (V = −0.8 V, I = 10 pA), respectively. The insets reveal the fast Fourier transform where the white circles are drawn as a guide to show the peaks associated with the atomic lattice. The blue circles in (**d**) are drawn as a guide to show the peaks associated with the Moiré lattice. The scale bars in (**c**) and (**d**) represent 3 nm. (**e**) A cartoon depicting the super modulation resulting from the top Sulfur atoms of MoS2 (S-S direction), and the top Carbon atoms of the HOPG (C-C direction) at a relative angle of 11 degrees. This misalignment of film and substrate produces a Moiré lattice with an 18 degree angle relative to the S-S direction of MoS_2_. (**f**) Scanning tunneling spectroscopy spectra averaged over several different locations per layer reveal a reduction in the bandgap with increasing layer number (set point: V = + 1.5 V, I = 200 pA). (**g**) Valence and conduction band edges in panel (f) are magnified to highlight their evolution with layer number.

The films prepared *ex-situ* were degassed at about 300 °C for about 10 hours in ultra-high vacuum to obtain clean surfaces suitable for STM investigation, and subsequently moved to the STM chamber without breaking the vacuum and cooled down at 4.2 K. Figure 2(a) and (b) show typical large-scale STM topographies of stacked MoS_2_ hexagonal sheets on HOPG. We find a uniform step height of the MoS_2_ islands of 0.7 nm, consistent with the c-axis lattice parameter of a single unit cell of MoS_2_^32^. Atomic resolution STM image taken on the substrate is shown in Fig. 2(c) where the lattice constant is determined to be 2.46 ± 0.02 Å in good agreement with the lattice parameter of HOPG^31^,^36^. Figure 2(d) shows an atomic resolution image acquired on monolayer MoS_2_ where the lattice constant is 3.13 ± 0.03 Å, in agreement with literature values^31^,^36^. We find that the atomic resolution image on the first layer MoS_2_ exhibits a superlattice structure (Moiré pattern), which results from lattice mismatch between the MoS_2_ film and the underlying HOPG. Occasionally, the S-S direction of the MoS_2_ is also rotated with respect to the underlying HOPG, as shown on Fig. 2(d). The fast Fourier transform shows inner peaks forming a hexagon (highlighted in blue in the inset of Fig. 2(d)) at an angle of 18° with respect to the primary peaks of the atomic lattice (white hexagon in the inset of Fig. 2(d)). This is consistent with the S-S direction of the MoS_2_ rotated by an angle of 11° with respect to the underlying HOPG lattice, as depicted in the schematic of Fig. 2(e).

In order to elucidate the thickness dependent electronic properties of MoS_2_, local STS measurements were performed on the first three layers in Fig. 2(a) and (b). The points at which the spectra were taken were carefully selected to be far from defect sites as well as the edges. The dI/dV curves shown in Fig. 2(f) were obtained by averaging 30 I-V curves per point before taking the derivative numerically. Several points per layer were then averaged. The edge of the valence band maximum (VBM) on the first MoS_2_ layer is located at 1.79 eV below the Fermi level (E_F_), and the conduction band minimum (CBM) is located at 0.27 eV above the E_F_, thereby yielding an intrinsic one-electron quasiparticle bandgap of 2.06 eV. The asymmetry of the spectra about E_F_ suggests that our films are n-doped, which is typical of films fabricated by CVD^29^,^31^,^37^. The spectra show a reduction of the band gap as the thickness increases. This reduction is mostly due to a shift of the valence band edge from −1.79 eV to −1.62 eV from the monolayer to the bilayer, while the transition from two to three layers presents a more subtle decrease. The conduction band edge remains fixed at +0.27 ±0.05 eV.

It has been reported from ARPES experiments and DFT calculations that the direct to indirect band gap transition occurs as a result of the valence band maximum shifting from the K-point to the Γ-point as the thickness is increased from one to two monolayers^18^. A change of the electronic band structure of atomically thin layers of MoS_2_ has also been reported and explained in terms of local strain^20^,^38^. We can exclude that the bandgap change obtained in STS measurements is due to local strain, as we do not observe a local change in the lattice parameter within our experimental resolution of about 1%. Any change in the lattice parameter below 1% could not account for the observed shift in the valence band edge of 0.17 eV. Our results are in very good agreement with the band structure calculations described below.

### X-ray photoemission spectromicroscopy and\mu-ARPES

**Figure 3:**
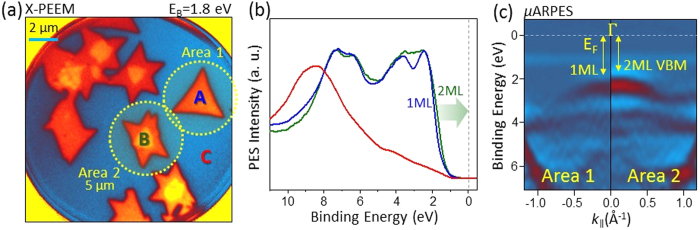
Valence band, spatially-resolved photoemission electron spectroscopy and μ-ARPES. (**a**) Spectromicroscopic investigation of the single- and double-layer MoS_2_ islands on HOPG. Kinetic-energy-filtered PEEM images are obtained with the photon energy of 100 eV. Photoelectron energy analyzer is tuned to the binding energy of 1.8 eV at which a clear photoemission intensity contrast is observed between the single- and double-layer MoS_2_ due to the difference in the bandgap size. (**b**) Spatially-resolved angle-integrated valence-band photoemission spectra for a single-layer MoS_2_ island (A), double-layer MoS_2_ area formed by two overlapping islands (B) and the HOPG substrate (C). Difference in the position of the VBM for the single- and double-layer MoS_2_ is consistent with changes in the bandgap size observed in STS. The HOPG spectrum intensity is scaled-down by a factor of two to enhance the visibility of the MoS_2_ spectra. (**c**) Momentum-resolved μ-ARPES spectra measured along the Γ-M high-symmetry direction at the locations labeled Area 1 and 2 in (**a**). Lateral resolution in the μ-ARPES measurement mode is approximately 5 μm, and for this reason some contribution from the HOPG substrate can be seen in the image. Strongly-hybridized Mo *d*_z_^2^ and S-*p*_z_ states, centered at the binding energy of approximately 2.3 eV at the Γ-point, show a clear broadening and shift towards E_F_ at the area containing the double-layer MoS_2_.

In order to investigate the spatially- and momentum-resolved valence-band electronic structure of the single- and double-layer MoS_2_ islands on HOPG, we utilized kinetic-energy-filtered x-ray photoemission microscopy (XPEEM) and micro-angle-resolved photoemission spectroscopy (μ-ARPES) at the UE49-PGM-a beamline at the BESSY-II storage ring (Helmholtz Zentrum Berlin). Figure 3(a) shows an XPEEM image acquired with the photon energy of 100 eV via valence-band photoelectron imaging at the binding energy of 1.8 eV, which corresponds to the rising edge of the valence-band maximum (VBM) of MoS_2_. A clear contrast between the single- and double-layer MoS_2_ islands at locations A and B is observed at this binding energy due to the difference in the bandgap size, which is evidenced by the 0.15 eV shift of the VBM towards the E_F_ in Fig. 3(b) and (c), as discussed below.

Location-resolved angle-integrated valence-band photoemission spectra collected at locations A and B with spatial resolution of <40 nm are shown in Fig. 3(b). These spectra provide a direct measure of the matrix-element-weighted density of states of the single- and double-layer MoS_2_ without detectable contribution from the HOPG substrate, which was measured separately at location C (shown in red). The absolute values of the VBM energies, determined within the uncertainty due to the combined beamline and analyzer resolution as well as the energy calibration, are in good agreement with the STS data.

Angle-resolved μ-ARPES spectra were measured along the Γ-M high-symmetry direction at the locations labeled Area 1 and 2 in Fig. 3(a) with spatial resolution of approximately 5 μm. These measurements provide complementary momentum-resolved information regarding the electronic structure of the single- and double-layer MoS_2_ islands, which is not accessible via STS and angle-integrated photoemission spectroscopy. Resulting second-derivative images containing spectral contributions from both the MoS_2_ islands as well as the HOPG substrate are shown in Fig. 3(c) and agree well with prior studies^39^,^40^. The hybridized Mo-*d*z_2_ and S-*p*_z_ states centered at the binding energy of approximately 2.3 eV straddle the Γ-point, and a clear shift of the VBM towards the E_F_ is observed for the double-layer MoS_2_ bands, in excellent agreement with our band-structure calculations described below. Measurement at the K point of the Brillouin zone is obscured by the intense π bands of the surrounding HOPG substrate^39^,^40^ and for this reason is not shown.

### Theoretical modeling and analysis

**Figure 4:**
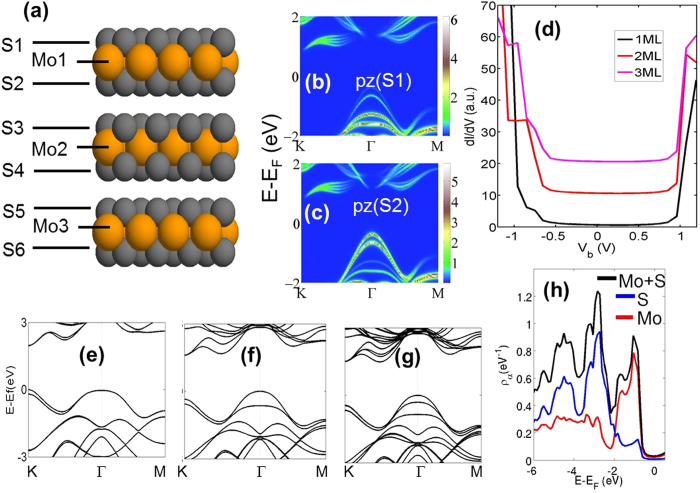
Calculated evolution of the electronic structure of mono- to few-layer MoS_2_. (**a**) Arrangement of layers in three-layer MoS_2_ film. The surface S-layer (S1) and the interfacial S-layers (S2 and S3) are marked. (**b**) and (**c**) Band dispersions giving weights of p_z_-orbitals of the surface and interfacial S atoms, respectively. (**d**) Theoretical dI/dV spectra for different layer thicknesses. (**e–g**) Band structures for one-, two and three-layer films, respectively. (**h**) Orbital-projected LDOSs from the two topmost layers of a 2 ML slab. Contributions from d-orbitals of Mo (red line), p-orbitals of S (blue line), and the sum of these two contributions (black line) are shown.

The computational studies of scanning tunneling spectroscopy/microscopy (STS/STM) and XPEEM/μ-ARPES experiments were performed within the framework of a realistic, Slater-Koster type tight-binding (TB) model Hamiltonian in which the overlap amplitudes were obtained through fits to first-principles band structures of one- and several-layer thick MoS_2_ films using WANNIER90^41^ and VASP codes^42^,^43^. Our TB Hamiltonian faithfully reproduces dispersions of ab initio valence and conduction bands and the associated wave functions and orbital characters. Special care was taken to ensure that our TB Hamiltonian correctly captures the evolution of the dispersions, orbital characters and spin-splittings around the VBM and the conduction band minimum (CBM) as the film transitions from a direct to an indirect gap with increasing thickness (Fig. 4(e–g)).

Our basic building block for modeling the geometry of the system is the two-dimensional (2D) primitive cell of a single MoS_2_ layer, which contains one Mo and two S atoms. An ABA stacking is used to construct multiple layers as shown in Fig. 4(a) for a three-layer film. Note that the environment of the surface S atoms (S1) differs from that of the interfacial S atoms (S2 and S3). Two spin degrees of freedom are included for each orbital with S atoms modeled with one s-orbital and three p-orbitals, and Mo with one s-orbital and five d-orbitals. As a result, our unit cell has 2 × 14, 2 × 28 and 2 × 42 total orbitals in 1, 2 and 3 layer MoS_2_ films, respectively. While these primitive cells are adequate for band structure calculations and orbital projections, a simulation cell of 4 × 4 unit cells was used in STM/STS calculations in order to model an adequately wide scanning area.

We cast the TB Hamiltonian in the following Slater-Koster^44^,^45^ form:


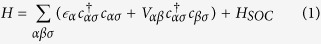


where *∈*_*α*_ is the onsite energy of orbital α, σ is the spin index and *V*_*αβ*_ is the hopping integral between orbitals α and β. For interlayer interactions we use slightly weakened hopping integrals to better model the three-layer film. *H*_*SOC*_ takes into account spin-orbit coupling (SOC) between the onsite Mo d-orbitals; the much weaker SOC of S atoms is neglected. The form of


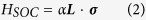


follows that of ref. 46, where ***L*** denotes angular momentum. We fit the only parameter α in Eq. (2) to reproduce the spin-orbit splitting of the ab-initio band structure.

The computation of the STS/STM spectra and interpretation of XPEEM results requires the construction of the Green’s function based on the Hamiltonian of Eq. (1). Many-body and external interactions can then be included in the form of self-energy matrices, Σ^47^,^48^,^49^,^50^,^51^:


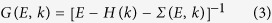


In the present calculations, we make the simplest possible approximation for an inelastic background, and use a term ∑ = −*iη*, where η is a small (real) positive parameter.

The Green’s function matrix of Eq. (3) is used to derive the density matrix:


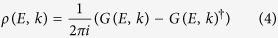


**Figure 5:**
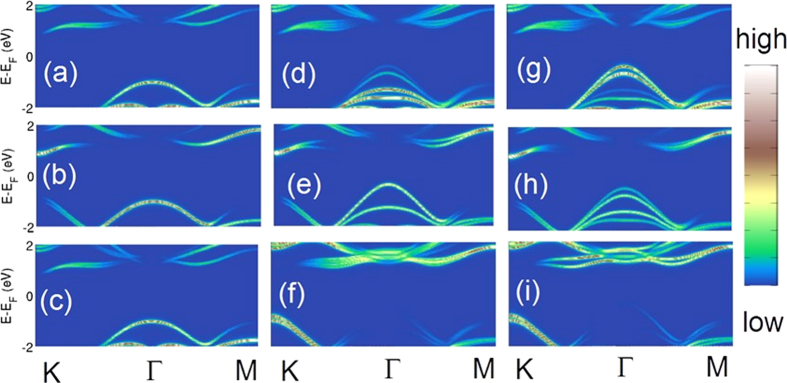
Contributions of various orbitals to the valence and conduction bands in MoS_2_ films along high-symmetry lines in the Brillouin zone. (**a–c**) Monolayer film. (**d–f**) Upper layer of the three-layer film. (**g–i**) Middle layer of the three-layer film. The upper row gives contribution of S-p_z_ orbital at three positions: the surface S-atom of one-layer film (**a**), the surface S-atom of three-layer film (**d**), and an interface S-atom of the three-layer film (**g**). The middle row shows the corresponding contributions of Mo-d_z_^2^ orbitals, and the last row of Mo-d_x2−y2_ orbitals.

The matrix elements, *ρ*_*ασβσ*_(*E,k*), of the density matrix are the key quantities that enter the computation of the spin/orbital projections of states and STM/STS spectra. In Fig. 4(b,c) and Fig. 5, the orbital projections are obtained by plotting the density matrix elements of given orbitals as a function of energy and momentum. In STM calculations, we assume that the HOPG substrate does not significantly affect the electronic structure of the MoS_2_ overlayers, and can therefore be neglected. The differential conductance between the tip and the sample is then given by^52^,^53^


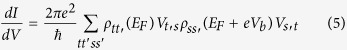


where ρ is the electron density matrix and *V*_*ts*_ is the hopping integral between a tip orbital *t* and a sample orbital *s*. The local density of states (LDOS) of the tip is assumed constant as is appropriate for an s-wave tip. This approach is related to the Tersoff-Hamann approximation^54^, and it has been used extensively in our earlier publications^51^,^55^,^56^. We turn now to interpret the experimental STS spectra in the light of our computations. A comparison of Figs 2(e) and 4(d) shows that the simulated *dI*/*dV* spectra follow the experimentally observed trends strikingly well. In particular, in both theory and experiment, increasing film thickness shifts the top of the valence band upward, while the bottom of the conduction band remains intact, and as a result, the band gap decreases. Insight is provided by our band structure computations, which indicate that the nature of the band gap changes abruptly as we go from one to two layer MoS_2_ film. In the one layer system, the band gap is direct at the K-point, but for two or more layers, the band gap becomes indirect as the valence bands move to higher energies at Γ-point, see Fig. 4(e–g).

The fundamental mechanism responsible for the dependence of the spectral gap on layer thickness becomes obvious when we consider contributions of different orbitals to the electronic states in the vicinity of the E_F_, see Fig. 5. In the one-layer film, the maximum of the valence band at the K point has a distinct Mo *d*_*xy*_ and *d*_*x2*−*y2*_ orbital character, while the states at the Γ-point have a strong Mo *d*_*z2*_-character mixed with S-*p*_*z*_ orbitals. In the two-layer system, on the other hand, the orbitals of interfacial S atoms overlap with each other, making the states at Γ-point rehybridize. These results are in accord with the tight-binding analysis of Cappeluti *et al*.^57^, who analyzed the orbital character/symmetry of interlayer hopping between the layers. Our computations give further insight into how interlayer overlap is reflected to the measured spectra as follows. The surface and interface S atoms contribute to different bands: the top of the valence band at the Γ-point now originates from the interfacial S and the adjoining Mo atoms, while the surface S atoms contribute only weakly to the top of the valence band. As a result the associated states essentially follow the dispersion of the valence band in the one-layer case, see Fig. 4(b) and (c). Although STS measurements do not directly probe the momentum dependence of the electronic spectrum, some information can be adduced from the nature and size of the band gap seen in the differential conductance spectrum (Fig. 4(d)). Since the tunneling current depends on the overlap *V*_*ts*_ between the tip and surface orbitals (see Equation 5), we expect that the tunneling matrix element between the valence states at the K-point and the tip will be relatively small compared to that for states at the Γ-point. The reason is that the Mo d_x2−y2_ and d_xy_ orbitals, which are characteristic of the states at K, are not as extended in the out-of-the-surface direction as the Mo-d_z2_ and S-p_z_ orbitals, which dominate the VBM states at the Γ-point. This qualitative analysis based on the symmetry of the atomic orbitals suggests that the measured valence band edges in the STS spectra here predominantly originate from the Γ-point.

For analyzing XPEEM and μ-ARPES experiments, we utilize density matrix as projected on the topmost S-layer and the Mo layer lying below the S-layer. We consider two different presentations of the density matrix derived from the Green’s function: 1) for the momentum-resolved μ-ARPES, we analyze the energy-momentum map of the density matrix projected on the two topmost atomic layers; 2) For angle-integrated XPEEM, a similar orbital projection of the LDOS is considered. More specifically, the LDOS-spectra are projected on to the d-orbitals of Mo and the p-orbitals of S on the two top layers. The orbital-projected band diagrams of Fig. 4 (b) and (c) indicate that the experimentally found shift of the VBM as we go from 1 ML to 2 ML originates from the intermediate S atoms. Note that the LDOS associated with the topmost S layer is small at the VBM, indicating that the dz^2^-orbitals of Mo play an important role mediated by the effects of the interlayer coupling.

In addition to the broadening of the VBM states at the Γ-point for increasing layer thickness, we have characterized features of the integrated XPEEM spectra by looking at the layer-resolved LDOS. While the peaks closest to the VBM display the strong *d*-character of Mo, there is a relatively wide energy range over which the spectral features are dominated by the p-character of S (see Fig. 4 (h)).

In order to gain insight into the μ-ARPES spectra, it is useful to classify the d-orbitals of Mo in terms of the absolute value of the magnetic quantum numbers, |m_l_|. Orbitals with |m_l_| = 1 give little contribution to valence bands, the |m_l_| = 2 orbitals strongly contribute to the spin-orbit-split states at the K-point, while the |m_l_| = 0 orbital is mainly responsible for the top of the valence band at the Γ-point. As already noted above, for the 1 ML film, the VBM lies at the K-point, but it moves to the Γ-point as the number of layers increases. In this connection, Fig. 5 shows |m_l_| = 0 ((c), (f), (I)) and |m_l_| = 2 ((b), (e), (h)) contributions to the band structure from Mo-d_x2−y2_ and Mo-d_z2_ orbitals. Figure 5 (d) and (e) show that the Γ-point signal indeed originates from dz^2^-orbitals of Mo, and less so from p_z_ of S. In contrast, Fig. 5 (e) and (f) indicate that the states around the K-point possess a stronger dx^2^−y^2^ compared to dz^2^ character.

**Figure 6:**
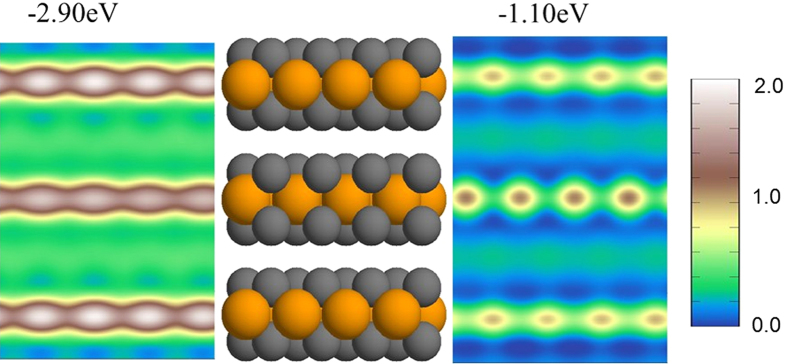
Local Density of States maps. Side view of a 3 ML film (middle). On the left and right side of the figure are shown spatially-resolved LDOS maps at two different binding energies, which correspond to the two large peaks in Fig. 4 (h).

Finally, Fig. 6 considers real space cross-sections at two different energies to gain a handle on the spatial distribution of the states related to spectral peaks in the XPEEM spectra. We see from Fig. 6 that the high amplitude states at −1.10 eV with p-character are less localized on the S-layers compared to the Mo layers and the space between the layers. At the higher binding energy of 2.90 eV (left panel in Fig. 6), the states are also mainly localized in the Mo layers, but there is also a significant spectral intensity at the interlayer space, which would make the related spectral features more susceptible to substrate effects.

## Conclusions

In this high-resolution STM/STS study, we have investigated the intrinsic electronic properties of atomically thin MoS_2_ layers directly grown on a graphite surface using the APCVD technique. The electronic band gap of the single layer MoS_2_ is determined to be 2.06 eV by STS spectra taken at 4.2 K, and it is suppressed by approximately 0.17 eV in the bilayer. The bandgap decrease is mostly due to a valence-band-edge shift as confirmed by photoemission microscopy and μ-ARPES measurements on the same samples. Parallel computational modeling of the electronic structure of the films and the associated STS spectra reveals that the interfacial S atoms are mainly responsible for the change in band structure and the observed shift of the valence band edge. Our study provides a comprehensive understanding of the intrinsic electronic properties of 2D TMD materials and impacts the development of new possibilities for electronic applications that utilize the control of two-dimensional layer thickness.

## Methods

### Film Growth

Mono- to few-layer thick MoS_2_ islands were grown using the well-known ambient pressure chemical vapor deposition technique using ultra-high-purity N_2_ (250sccm) as the carrier gas. HOPG substrates were cleaved with scotch tape just prior to loading in the furnace. Substrates were suspended facedown above ~15 mg of MoO_3_ (≥99.5% Sigma Aldrich) in a crucible placed downstream from a different crucible containing 80 mg of Sulfur (≥99.5% Sigma Aldrich). Each crucible was placed in a different heating zone in a 1” furnace. Temperatures in these two zones were individually controlled using two adjacent tube furnaces. The furnace containing the MoO_3_ and HOPG was degassed at 150 °C for 90 minutes then ramped to 700 °C at a rate of 15 °C/min. Once this furnace reached 320 °C the furnace containing the Sulfur crucible was ramped to 120 °C at approximately 3 °C/min. Both furnaces were allowed to sit at their maximum temperatures for 30 minutes at which point the MoO3 furnace was ramped down at 8 °C/min. Once this furnace reached 580 °C both furnaces were rapidly cooled to room temperature.

### STM/STS

Scanning tunneling microscopy and spectroscopy measurements were carried out using a Unisoku STM with PtIr tip at T = 4.2 K in an ultra-high vacuum (<10^−11^ Torr) at T = 4.2 K. Prior to measurements all samples were degassed at approximately 300 °C and 10^−10^. Torr for a minimum of three hours up to ten hours and subsequently moved to the scanner without breaking the vacuum. The STM images were recorded in constant current mode with tunneling current of 10–250 pA. For the *dI*/*dV* spectra an average of 30 I-V curves were acquired at each location, and curves from different locations within the same layer were averaged to obtain the *dI*/*dV* conductance spectra by numerical derivative.

### XPEEM and μ-ARPES

X-ray photoemission spectromicroscopy and μ-ARPES measurements were performed at the SPEEM station located at the UE49/PGMa beamline at BESSY-II (Helmholtz Zentrum, Berlin). The instrument is based on the Elmitec III photoemission electron microscope with an integrated electrostatic photoelectron energy analyzer between the objective column and the projective lenses. The analyzer functions as the kinetic energy filter thereby enabling spectromicroscopic imaging via core-level and valence-band photoelectrons, which was the key capability utilized in this study. The photon energy was fixed at 100 eV and the analyzer energy was varied across the range corresponding to the MoS_2_ valence-band manifold. Typical lateral resolution facilitated by the instrument in the PEEM mode is 30 nm. An aperture in the first image plane of the microscope was used to select the location on the sample for angle-resolved valence-band spectroscopy (μ-ARPES) measurements. The minimum size of the selected area is approximately 5 μm, which defines the spatial resolution in the μ-ARPES mode. Prior to the measurement, the sample was annealed *in-situ* at 300 °C for 3 hours in order to eliminate surface adsorbates. All measurements were carried out at 20 K.

### AFM

Atomic force microscopy images were acquired in tapping mode using a Veeco Dimension Icon SPM with an Antimony (n) doped Si tip having a nominal tip radius of 10 nm (Veeco, NCHV). In this mode, the cantilever is driven to oscillate at its resonance frequency of 320 Hz by applying an AC voltage to the z-piezo. As long as the tip is far away from the sample, no interaction is recorded and the oscillation amplitude remains constant. Once the cantilever is moved closer to the sample, the tip starts touching the surface intermittently. As a consequence, changes in the cantilever oscillation amplitude are induced by the Van der Waals interaction. In such a scenario, the amplitude modulations recorded while scanning on the surface are caused by the sample roughness. Here, a feedback loop is used to fix the tip-sample separation point-by-point in order to keep the amplitude constant. The adjustments in tip-sample distance, driven by the feedback loop, are thus a measure of sample topography. AFM measurements were carried out under ambient conditions.

### Note

^†^E.R. Caianiello Physics Department and NANOMATES, Research Centre for Nanomaterials and Nanotechnology, University of Salerno, Fisciano (SA), Italy.

## Additional Information

**How to cite this article**: Trainer, D. J. *et al*. Inter-Layer Coupling Induced Valence Band Edge Shift in Mono- to Few-Layer MoS_2_. *Sci. Rep.* **7**, 40559; doi: 10.1038/srep40559 (2017).

**Publisher's note:** Springer Nature remains neutral with regard to jurisdictional claims in published maps and institutional affiliations.
