# Erratum: Inter-Layer Coupling Induced Valence Band Edge Shift in Mono- to Few-Layer MoS_2_

**DOI:** 10.1038/srep42619

**Published:** 2017-03-14

**Authors:** Daniel J. Trainer, Aleksei V. Putilov, Cinzia Di Giorgio, Timo Saari, Baokai Wang, Mattheus Wolak, Ravini U. Chandrasena, Christopher Lane, Tay-Rong Chang, Horng-Tay Jeng, Hsin Lin, Florian Kronast, Alexander X. Gray, Xiaoxing Xi, Jouko Nieminen, Arun Bansil, Maria Iavarone

Scientific Reports 7: Article number: 4055910.1038/srep40559; published online: 01 13 2017; updated: 03 14 2017

The original version of this Article contained an error in the spelling of the author Xiaoxing Xi, which was incorrectly given as Xiaoxing X. Xi.

In addition, the Article contained a typographical error in the Results section. The subheading,

“X-ray photoemission spectromicroscopy and\mu-ARPES”

now reads:

“X-ray photoemission spectromicroscopy and μ -ARPES”.

These errors have now been corrected in the PDF and HTML versions of the Article.

